# Pyroptosis by NLRP3/caspase‐1/gasdermin‐D pathway in synovial tissues of rheumatoid arthritis patients

**DOI:** 10.1111/jcmm.17834

**Published:** 2023-06-29

**Authors:** Xue Zhang, Qiuyuan Wang, Guorui Cao, Manli Luo, Hongli Hou, Chen Yue

**Affiliations:** ^1^ Department of Rheumatology Luoyang Orthopaedic Hospital of Henan Province Luoyang China; ^2^ Department of Orthopaedic Surgery Luoyang Orthopaedic Hospital of Henan Province Luoyang China

**Keywords:** caspase‐1, gasdermin D, NLRP3, pyroptosis, rheumatoid arthritis, synovial tissue

## Abstract

We investigated the potential involvement of pyroptosis, a proinflammatory form of regulated cell death, in rheumatoid arthritis (RA). Synovial fluid, synovial tissues and/or serum were compared among 32 patients with RA, 46 patients with osteoarthritis (OA) and 30 healthy controls. Samples were assayed for interleukin (IL)‐1β, IL‐18 and lactate hydrogenase (LDH). Synovial expression of NLRP3, caspase‐1 and cleaved gasdermin D (GSDMD) was assayed using immunohistochemistry and multiplex immunohistochemistry. Patients with RA showed significantly higher levels of IL‐1β and IL‐18 in synovial fluid than patients with OA, and significantly higher levels of both cytokines in serum than healthy controls. RA was associated with higher levels of LDH in synovial fluid than OA. Among patients with RA, levels of IL‐1β, IL‐18 and LDH were significantly higher in synovial fluid than in serum, and the levels in synovial fluid positively correlated with disease activity and inflammation. Synovial cells, particularly macrophages, showed upregulation of NLRP3, caspase‐1 and cleaved GSDMD in RA compared to OA. Our results implicate pyroptosis in the pathogenesis of RA, perhaps as a driver of local inflammation in joints.

## 1 INTRODUCTION

Rheumatoid arthritis (RA), a chronic inflammatory joint disease, can lead to irreversible disability if untreated,^1^ and current treatments fail to achieve remission in many patients.^2^ Therefore, further insights into pathogenesis of the disease are urgently needed in order to guide the search for novel therapeutic targets.

Recent evidence suggests that pyroptosis, a newly discovered type of regulated cell death, may contribute to RA. In pyroptosis, the NLRP3 inflammasome activates caspase‐1,^3^, ^4^ which in turn activates pro‐inflammatory cytokines such as interleukin (IL)‐1β and IL‐18. Caspase‐1 as well as other caspases can cleave gasdermin D (GSDMD), and the GSDMD‐N‐terminal (GSDMD‐N) creates pores in the plasma membrane leading to leakage of contents such as lactate dehydrogenase (LDH).^5^, ^6^, ^7^ Knockout of NLRP3 ameliorated joint injury and attenuated inflammatory‐response in a collagen‐induced arthritis mice model.^8^ Deleting caspase‐1 from a mouse model of chronic arthritis mitigated joint inflammation and cartilage destruction,^9^ and caspase‐1 activation appears to be common in T cells resident in the lymph nodes of patients with RA.^10^ Exposing monocyte cultures to serum from patients induced GSDMD‐dependent pyroptosis, which correlated with disease activity.^11^

Here we explored the implication of pyroptosis in RA by comparing levels of IL‐1β, IL‐18 and LDH in serum or synovial fluid among patients with RA, patients with osteoarthritis (OA) and healthy controls. We also examined expression of NLRP3, caspase‐1 and GSDMD‐N in the synovium. Necroptosis is another type of regulated cell death, and caspase‐1 does not participate in the process. Moreover, IL‐1β and IL‐18 are long considered signatures of pyroptotic cell death and GSDMD executes pyroptosis.^12^ These indicators help to distinguish pyroptosis from necroptosis. Since OA arises from mechanical injury and involves milder inflammation and tissue damage than RA,^13^ we compared synovium from patients with OA to that from patients with RA. We hope, through the human specimens, to provide clinical evidence of the role of pyroptosis in RA.

## 2 METHODS

### 2.1 Samples

The study protocol was approved by the ethics committee of Luoyang Orthopaedic Hospital (KY2022‐001‐02). Synovial tissue and synovial fluid were sampled from 32 patients with RA and 46 patients with OA who underwent knee arthroplasty in our department of orthopaedic surgery between October 2020 and June 2022. RA was diagnosed based on the 2010 criteria of the American College of Rheumatology and European League Against Rheumatism,^14^ while OA was diagnosed based on the most recent criteria of the European League Against Rheumatism.^15^ In addition, we collected serum from the 32 patients with RA on the day before surgery, as well as from 30 healthy controls who visited our clinic for routine outpatient exams. We excluded subjects who received oral prednisolone ≥10 mg daily or a glucocorticoid injection within the previous 3 months.^16^ All participants signed written informed consent before participating in the study.

The following clinical data were prospectively collected from patients as appropriate: clinicodemographic characteristics, C‐reactive protein (CRP), erythrocyte sedimentation rate (ESR) and score on the ‘Disease Activity Score in 28 Joints‐ESR’ (DAS28‐ESR) scale.^17^

### 2.2 Analysis of IL‐1β, IL‐18 and LDH

Commercial kits were used to assay synovial fluid and serum for IL‐1β (catalogue no. EHC002b, Neobioscience) and IL‐18 (EHC127, Neobioscience) according to the manufacturer's instructions. Samples were incubated for 90 min in 96‐well plates that had already been precoated with the desired antibody. The plate was washed, incubated with biotinylated detection antibody for 60 min, washed again, incubated with enzyme conjugate for 30 min, washed again and incubated with chromogenic substrate followed by stop solution. The LDH level in synovial fluid and serum was assayed using a commercial kit (A020‐2, Nanjing Jiancheng Bioengineering Institute) according to the manufacturer's instructions. In all assays, absorbance at 450 nm was measured using a microplate reader (BioTek,).

### 2.3 Histopathology

Synovial tissue was fixed with 4% paraformaldehyde, embedded in paraffin, sliced to a thickness of 4 μm, and stained with haematoxylin and eosin. Tissues were viewed under a BX40 light microscope (Olympus) and photographed using a digital camera (Olympus), and histopathology was assessed as described.^18^ The average number of cells per 1 mm^2^ was determined from three randomly selected fields at a magnification of 400×. The thickness of the synovial lining layer was determined by averaging the number of cells in three fields randomly selected in longitudinal tissue sections at a magnification of 400 × .

### 2.4 Immunohistochemistry

Paraffin‐embedded synovial tissues were also analysed using immunohistochemistry. Sections were deparaffinized in xylene, rehydrated in ethanol, microwaved in citrate buffer to retrieve antigens, incubated in 3% H_2_O_2_ for 10 min followed by goat serum for 60 min at room temperature, and then incubated overnight at 4°C with antibodies against NLRP3 (1:100 dilution; catalogue no. MAB7578, R&D, Minneapolis, MN, USA), caspase‐1 (1:100; MAB62154, R&D) or GSDMD‐N (1:500; 36,425 s, CST,). Next, sections were incubated for 30 min at room temperature with horseradish peroxidase‐linked goat secondary antibodies against rat IgG, mouse IgG or rabbit IgG (CST), followed by treatment with 3,3′‐diaminobenzidine (catalogue no. SK4100, Vector Labs,) and Mayer's haematoxylin. Stained sections were viewed under a BX40 light microscope (Olympus).

### 2.5 Multiplex immunohistochemistry

Paraffin‐embedded synovial tissue sections were subjected to multiplex immunohistochemistry. These sections were deparaffinized, rehydrated and incubated in 3% H_2_O_2_ as described above, then washed in 1× Tris‐buffered saline containing 0.5% Tween‐20 (TBST) and subjected to multiplex staining using a commercial kit (catalogue no. NEL861001KT, Akoya Bioscience) according to the manufacturer's instructions. Sections were subjected to antigen retrieval, blocked in blocking solution at room temperature, then incubated with primary antibody against the macrophage marker CD68 (1:800 dilution; catalogue no. 76437, CST) or with the same primary antibodies described above against NLRP3, caspase‐1 or GSDMD‐N. Sections were rinsed in TBST, incubated with horseradish peroxidase‐conjugated secondary antibody and incubated with Opal. The once‐stained sections were again subjected to antigen retrieval following by staining with the subsequent antibody in the multiplex as described above. Finally, nuclei were stained with 4,6‐diamidino‐2‐phenylindole (DAPI), sections were washed with TBST and fluorescent anti‐quencher was added. Multispectral fluorescence images were acquired using the Vectra Polaris Automated Quantitative Pathology Imaging System (Akoya Biosciences).

## 3 STATISTICAL ANALYSIS

Data were reported as mean ± SD and analysed statistically using SPSS 22.0 (IBM, Chicago, IL, USA). Intergroup differences in continuous variables were assessed for significance using Student's *t* test or the Mann–Whitney test. Differences in categorical variables were assessed using a chi‐squared test. Potential associations between variables were analysed using bivariate Pearson correlation. Differences associated with *p* < 0.05 were considered statistically significant.

## 4 RESULTS

### 4.1 Characteristics of patients and controls

**FIGURE 1:**
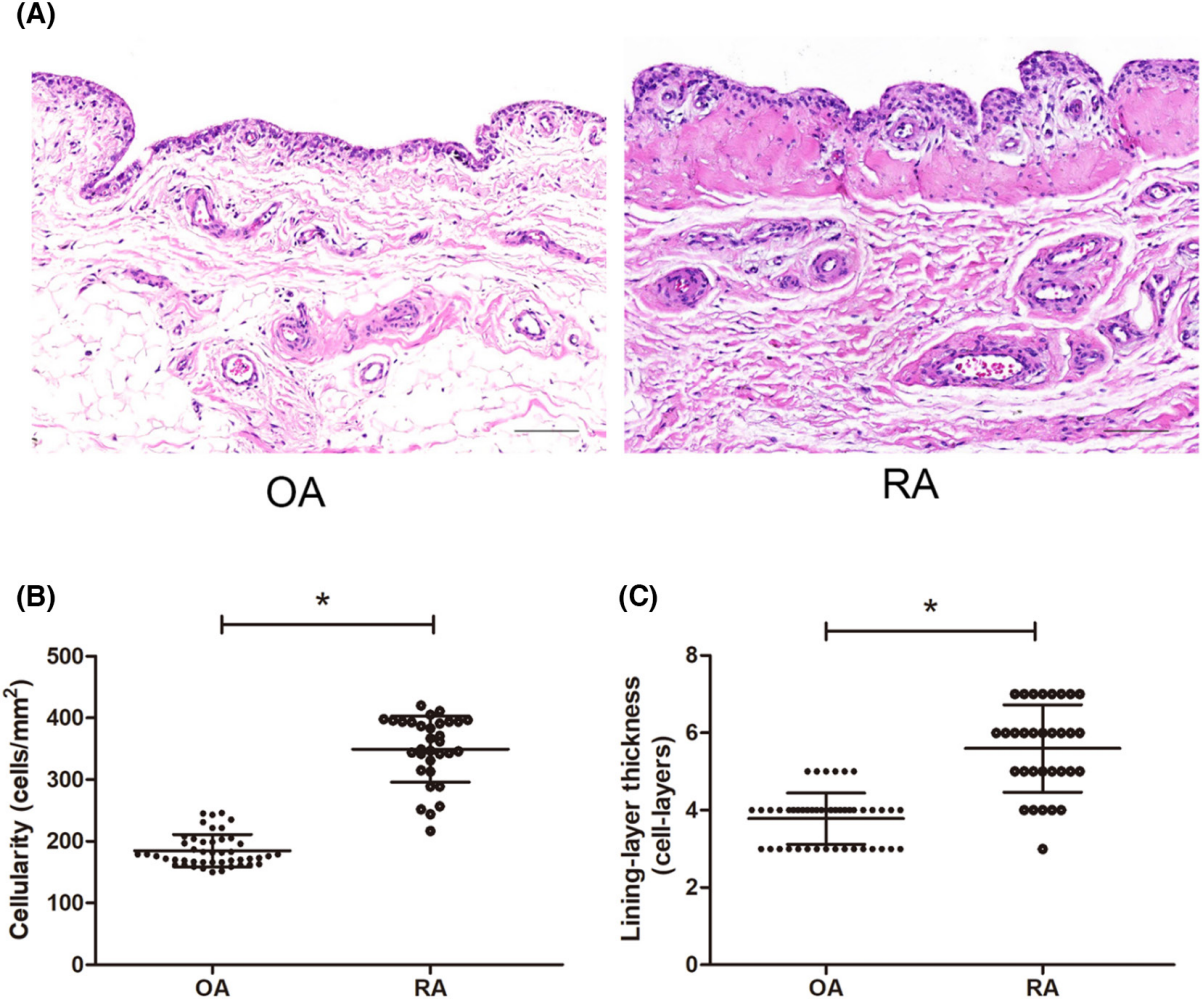
Tissues from patients with osteoarthritis (OA) or rheumatoid arthritis (RA) were stained with haematoxylin and eosin. (A) Representative micrographs. Scale bar, 100 μm. (B) Quantitation of synovial cell density. (C) Quantitation of the thickness of the lining layer. * *p* < 0.001.

Characteristics of the study participants are summarized in Table 1. The two groups of patients and the healthy controls were similar in sex distribution and age. Patients with RA showed significantly higher levels of CRP and ESR in blood than patients with OA and healthy controls. Patients with RA had a mean rheumatoid factor (RF) level of 42.5 IU/m and mean DAS28‐ESR score of 3.3, and their synovial cell density and lining‐layer thickness were significantly greater than those of patients with OA (Figure 1A,B).

**TABLE 1:** Characteristics of patients with rheumatoid arthritis, patients with osteoarthritis and healthy controls.

Variables	RA (*n* = 32)	OA (*n* = 46)	Normal (*n* = 30)
Sex (Male/female)	2/30	9/37	3/27
Age (years)	62.5 ± 5.5	67.3 ± 5.2	63.7 ± 5.3
Duration of disease (years)	11.4 ± 6.4	9.6 ± 4.5	
CRP (mg/L)	24.0 ± 22.0	3.9 ± 2.7*	3.3 ± 2.6*
ESR (mm/h)	37.4 ± 29.1	13.5 ± 7.5*	10.2 ± 3.9*
RF (IU/ml)	42.5 ± 31.2		
DAS28‐ESR	3.3 ± 0.9		

*Note*: Data are expressed as mean ± standard deviation (SD); *p<0.05 vs. RA group.

Abbreviations: CRP, c‐reactive protein; DAS28‐ESR, disease Activity Score in 28 joints‐erythrocyte sedimentation rate; ESR, erythrocyte sedimentation rate; OA, osteoarthritis; RA, rheumatoid arthritis; RF, rheumatoid factor.

### 4.2 Pyroptosis‐related cytokines and LDH

**FIGURE 2:**
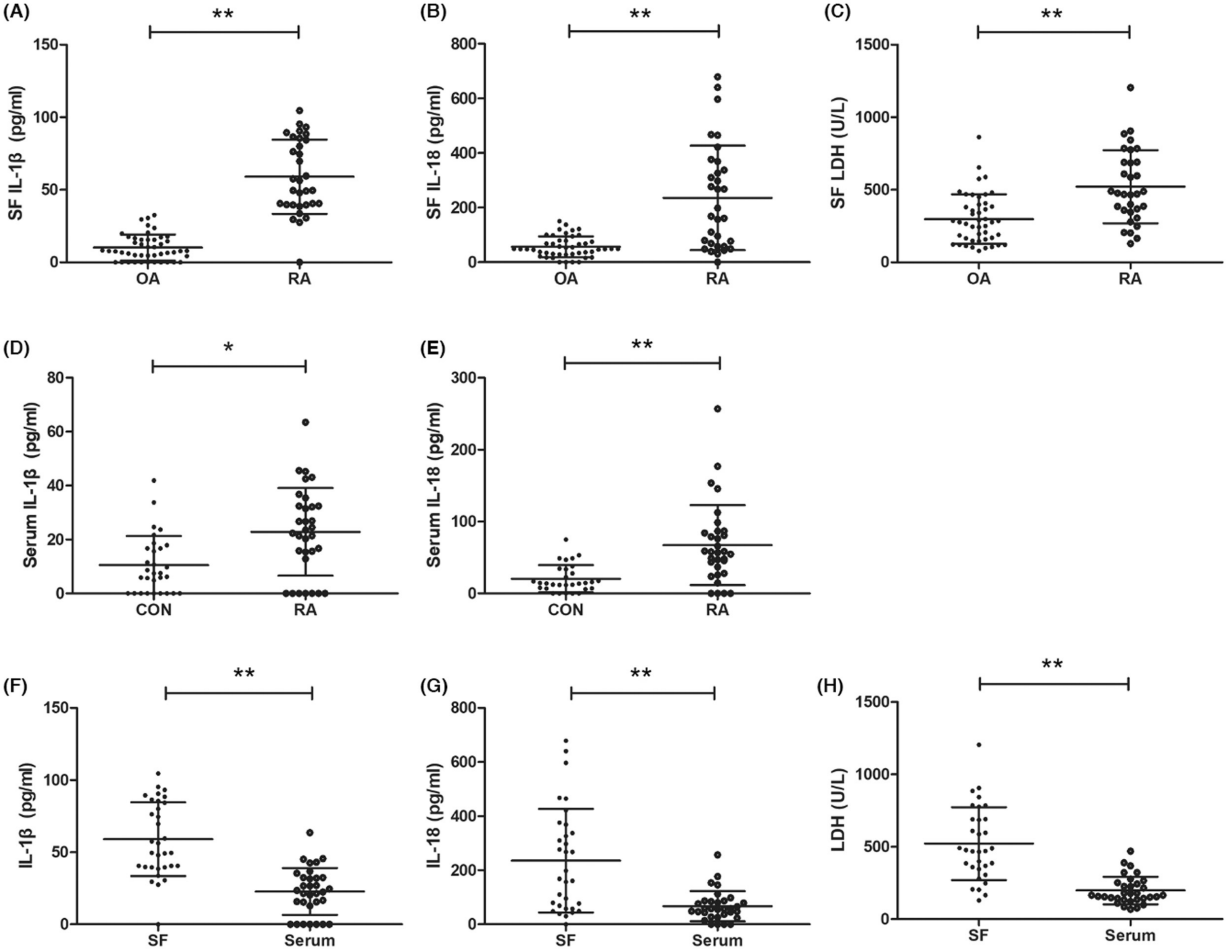
Levels of pyroptosis‐related cytokines and lactate dehydrogenase (LDH) in patients with rheumatoid arthritis (RA), patients with osteoarthritis (OA), or healthy controls (CON). (A–C) Assays of synovial fluid (SF). (D, E) Assays of serum. (F–H) Comparisons of marker levels between SF and serum. * *p* < 0.01, ** *p* < 0.001.

Patients with RA showed significantly higher levels of IL‐1β and IL‐18 in synovial fluid than patients with OA (Figure 2A,B), and they showed significantly higher levels of both cytokines in serum than healthy controls did (Figure 2D,E). Among patients with RA, levels of both cytokines were significantly higher in synovial fluid than in serum (Figure 2F,G). Levels of the cell death leakage marker LDH in patients with RA were significantly higher than levels in patients with OA based on analysis of synovial fluid (Figure 2C), yet similar to levels in healthy controls based on analysis of serum. Among patients with RA, LDH levels were significantly higher in synovial fluid than in serum (Figure 2H). These results are consistent with a local articular increase in pyroptosis in RA.

### 4.3 Associations between pyroptosis markers and clinical characteristics of RA

**FIGURE 3:**
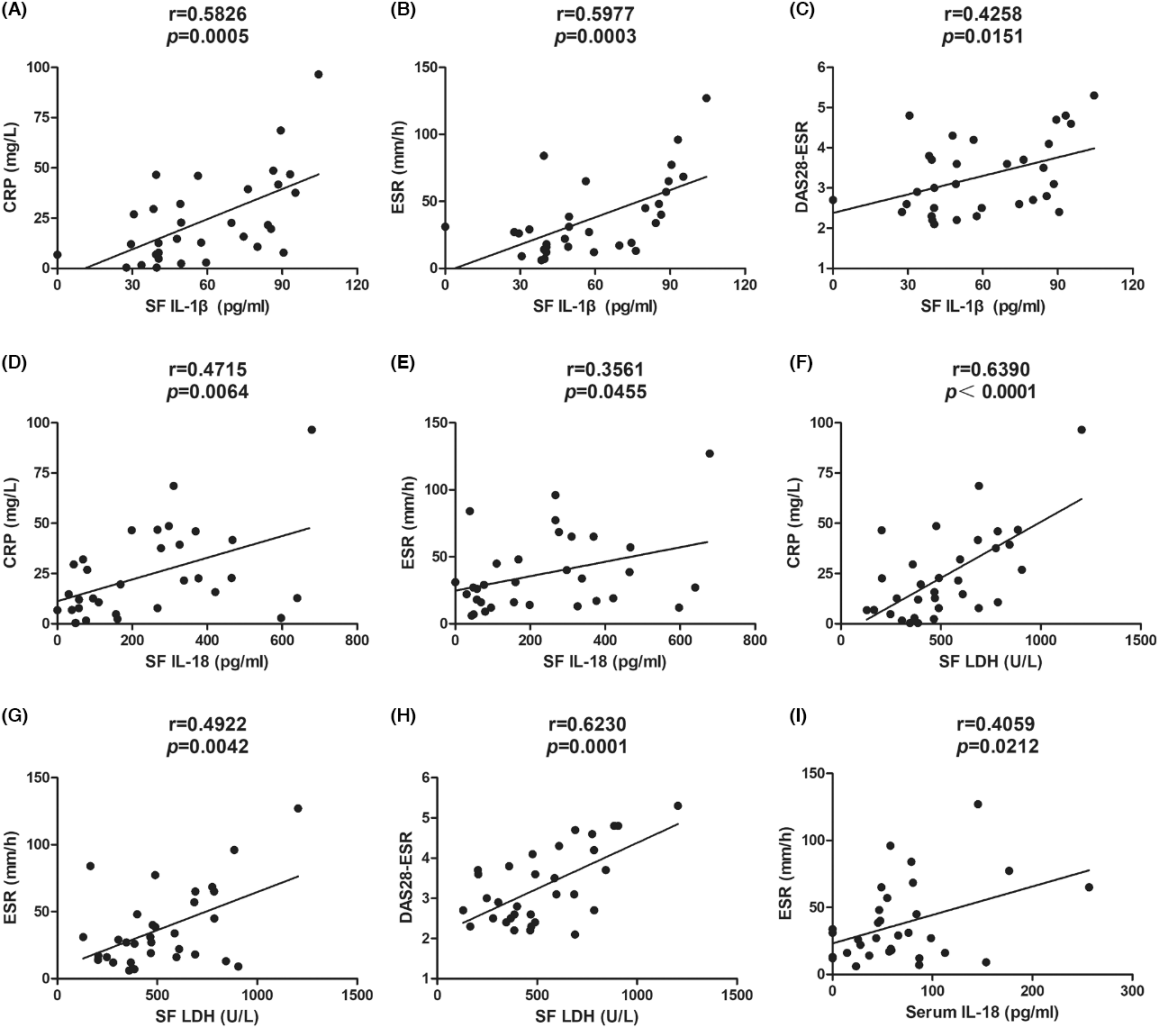
Correlation of clinical characteristics with (A–C) levels of IL‐1β in synovial fluid (SF), with (D, E) levels of IL‐18 in SF, with (F–H) lactate dehydrogenase (LDH) in SF, or with (I) levels of IL‐18 in serum of patients with rheumatoid arthritis. CRP, C‐reactive protein; ESR, erythrocyte sedimentation rate; IL, interleukin；DAS28‐ESR，Disease Activity Score in 28 Joints‐ESR.

In synovial fluid, levels of IL‐1β correlated positively with CRP level (*r* = 0.5826, *p* = 0.0005, Figure 3A), ESR (*r* = 0.5977, *p* = 0.0003, Figure 3B) and score on the DAS28‐ESR (*r* = 0.4258, *p* = 0.0151, Figure 3C), but not with level of RF. Levels of IL‐18 also correlated positively with CRP (*r* = 0.4715, *p* = 0.0064, Figure 3D) and ESR (*r* = 0.3561, *p* = 0.0455, Figure 3E), but they did not correlate with score on the DAS28‐ESR or RF level. Levels of LDH correlated positively with CRP (*r* = 0.6390, *p* < 0.0001, Figure 3F), ESR (*r* = 0.4922, *p* = 0.0042, Figure 3G) and score on the DAS28‐ESR (*r* = 0.6230, *p* = 0.0001, Figure 3H), but not with RF level.

In serum, the only significant association detected was a positive correlation between levels of IL‐18 and ESR (*r* = 0.4059, *p* = 0.0212, Figure 3I).

### 4.4 Associations between RA and synovial expression of NLRP3, caspase‐1 and GSDMD‐N

**FIGURE 4:**
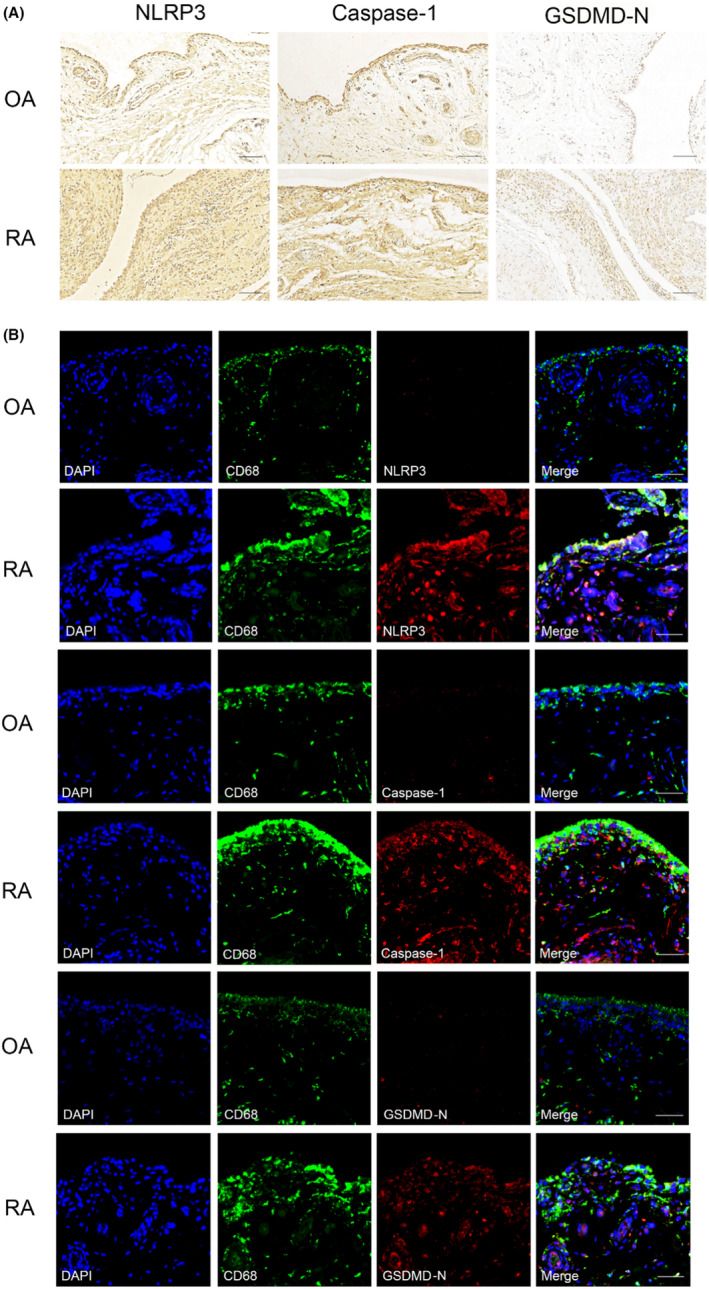
Expression of NLRP3, caspase‐1 and gasdermin D‐N‐terminal (GSDMD‐N) in synovial tissues from patients with osteoarthritis (OA) or rheumatoid arthritis (RA). (A) Sections were stained with antibodies against the indicated proteins. Scale bar, 100 μm. (B) Sections were stained with an antibody against the macrophage marker CD68 and another antibody against a pyroptosis‐related marker. Nuclei were counterstained with DAPI. Scale bar, 50 μm.

Immunohistochemistry and multiplex immunohistochemistry revealed upregulation of NLRP3, caspase‐1 and GSDMD‐N in the synovium of patients with RA compared to the corresponding tissue from patients with OA (Figure 4A,B). This upregulation was observed in various types of synovial cells, especially macrophages, which we identified using the marker CD68 (Figure 4B). Pyroptosis is known to occur mainly in macrophages and other phagocytes of the myeloid lineage, and the pathogenesis of RA is known to be driven by macrophages.^6^, ^19^ Our results are consistent with the increasement of synovial pyroptosis in RA which may create a hyper‐inflammatory microenvironment around joints.

## 5 DISCUSSION

**FIGURE 5:**
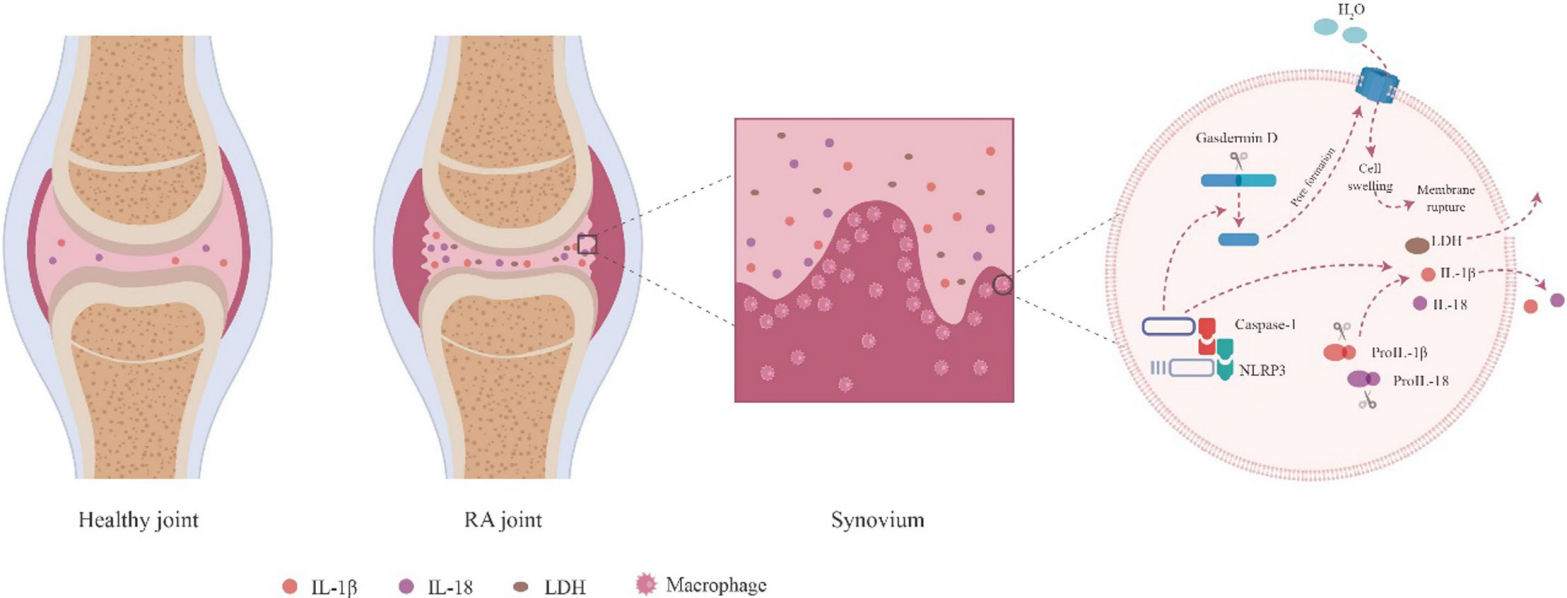
Schematic showing how pyroptosis may contribute to the pathogenesis of rheumatoid arthritis. In synovial cells, particularly macrophages, activation of the NLRP3/caspase‐1/gasdermin D pathway leads to pyroptosis. Caspase‐1 activates IL‐1β and IL‐18, which are released into the synovial fluid, while gasdermin D forms pores in the plasma membrane, leading to cytoplasmic swelling and ultimately to lysis, which releases lactate dehydrogenase and other cell contents into the synovial fluid.

Our comparison of samples from patients with RA, patients with OA and healthy controls suggests that RA is associated with local upregulation of pyroptosis in the synovium, which leads to the excessive release of inflammatory factors IL‐1β and IL‐18, which may in turn contribute to joint damage (Figure 5). Our finding of similar serum concentrations of LDH between patients with RA and healthy controls, despite the higher serum concentrations of IL‐1β and IL‐18 in patients, suggests that the increased pyroptosis is localized to joints. Our results imply that inhibiting this pyroptosis, perhaps through targeting of macrophages, may have therapeutic efficacy against the disease.

Consistent with our findings, synovial fluid levels of IL‐1β and IL‐18 are higher in patients with RA than in patients with OA in other studies.^20^, ^21^ IL‐1β contributes to the pathogenesis of RA by activating macrophages and monocytes to promote proliferation of fibroblasts, leading to synovial hyperplasia^22^; while IL‐18 contributes to the pathogenesis by inducing osteoclast formation.^23^ By flooding joint areas with these pro‐inflammatory cytokines, pyroptosis may help drive the disease.^24^ Indeed, we found that levels of IL‐1β, IL‐18 and LDH in synovial fluid correlated positively with CRP levels, ESR and DAS28‐ESR score. The levels did not, however, correlate with levels of RF, which is consistent with the unreliability of RF as a marker of RA activity.^25^

In the synovium of collagen‐induced arthritis mice, NLRP3 expression was positively correlated with arthritis severity.^26^ Antigen‐induced arthritis mice showed severe joint inflammation with increased expression of IL‐1β and NLRP3 inflammasome in synovium.^27^ Monomer derivative of paeoniflorin has a therapeutic effect on adjuvant arthritis rats by decreasing the ratio of macrophage pyroptosis via TLR4/NLRP3/GSDMD signalling pathway.^28^ These studies provide evidence in vivo and in vitro that NLRP3 inflammasome and GSDMD are involved in the pathogenesis of RA. Our experiments support the involvement of pyroptosis mediated by the NLRP3 inflammasome, caspase‐1 and GSDMD^29^ in RA, providing clinical support for similar results obtained with rat models of RA.^28^, ^30^ Our results may help explain the therapeutic effects of tofacitinib, a clinically licensed drug that works against the disease by inhibiting activation of the NLRP3 inflammasome.^31^ Other pyroptosis pathways may also contribute to RA. For example, expression of activated caspase 3 and gasdermin E in monocytes and synovial macrophages is higher in patients with RA than in patients with OA, and tumour necrosis factor induces pyroptosis in monocytes and macrophages by activating the caspase‐3/gasdermin E pathway.^32^ Further research should examine the range of pathways that hyperactivate pyroptosis in RA, as well as explore the various types of synovial cells, apart from macrophages, in which pyroptosis may be upregulated. In particular, increased GSDMD‐mediated pyroptosis in fibroblast‐like synoviocytes of patients with RA should be investigated.^33^ The mIHC staining showed that CD68 colocalized better with NLRP3 in compare with caspase‐1 and GSDMD‐N. NLRP3 can be upregulated through the recognition of various pathogen‐associated molecular patterns (PAMPs) or damage‐associated molecular patterns (DAMPs), and the activation of caspase‐1 needs the induction of posttranslational modifications of NLRP3 and some types of NLRP3 activation do not promote pyroptosis.^34^ We speculate it may be that some PAMPs or DAMPs may upregulate NLRP3 but not promote pyroptosis in macrophages.

Our work, together with the literature, suggests that inhibiting pyroptosis in the synovium may be an effective treatment against RA. Future work should clarify the range of cell types and signaling pathways involved, which may guide the development of specific therapies with minimal off‐target effects.

## AUTHOR CONTRIBUTIONS

**Xue Zhang:** Conceptualization (supporting); data curation (supporting); funding acquisition (equal); project administration (supporting); software (equal); writing – original draft (lead). **Qiuyuan Wang:** Investigation (equal); methodology (equal); project administration (supporting). **Guorui Cao:** Investigation (equal); methodology (equal); project administration (equal). **Manli Luo:** Investigation (equal); methodology (equal); project administration (equal). **Hongli Hou:** Methodology (equal); software (equal). **Chen Yue:** Conceptualization (lead); data curation (lead); funding acquisition (equal); project administration (lead); supervision (lead); validation (lead); writing – review and editing (lead).

## FUNDING INFORMATION

This work is supported in China by National Natural Science Foundation of China (82004396); Project of science and technology of the Henan province (212102311089); Heluo Youth Talent Promotion Project (2022HLTJ15).

## CONFLICT OF INTEREST STATEMENT

The authors declare no conflict of interest.

## Supporting information


**Figure S1:** Immunohistochemical staining of rat (A and D), mouse (B and E) or rabbit (C and F) antibody IgG isotype control for synovial tissues from patients with osteoarthritis (OA) or rheumatoid arthritis (RA). Scale bar, 100 μm.
Figure S2: Multiplex immunohistochemical staining of antibody IgG isotype control for synovial tissues from patients with osteoarthritis (OA) or rheumatoid arthritis (RA). Scale bar, 50 μm.

## Data Availability

Data of the study was available upon reasonable request from the corresponding author.
