# Supplementary material for: Pyroptosis by NLRP3/caspase‐1/gasdermin‐D pathway in synovial tissues of rheumatoid arthritis patients
Source: J Cell Mol Med. 2023 Jun 29;27(16):2448–56. doi: 10.1111/jcmm.17834 (PMC10424297; doi:10.1111/jcmm.17834)
Supplement: Supplementary file 1 — Figure S1: Immunohistochemical staining of rat (A and D), mouse (B and E) or rabbit (C and F) antibody IgG isotype control for synovial tissues from patients with osteoarthritis (OA) or rheumatoid arthritis (RA). Scale bar, 100 μm. Figure S2: Multiplex immunohistochemical staining of antibody IgG isotype control for synovial tissues from patients with osteoarthritis (OA) or rheumatoid arthritis (RA). Scale bar, 50 μm. [file JCMM-27-2448-s001.docx]

**Supplemental Figures**


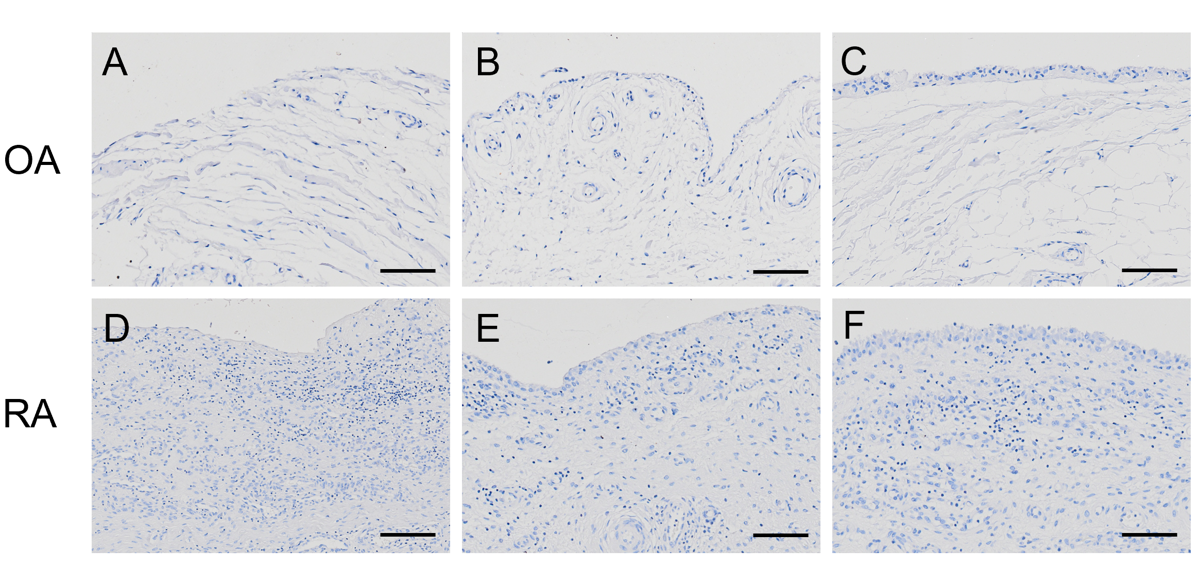


Figure S1: Immunohistochemical staining of rat (A and D), mouse (B and E) or rabbit (C and F) antibody IgG isotype control for synovial tissues from patients with osteoarthritis (OA) or rheumatoid arthritis (RA). Scale bar, 100 μm.


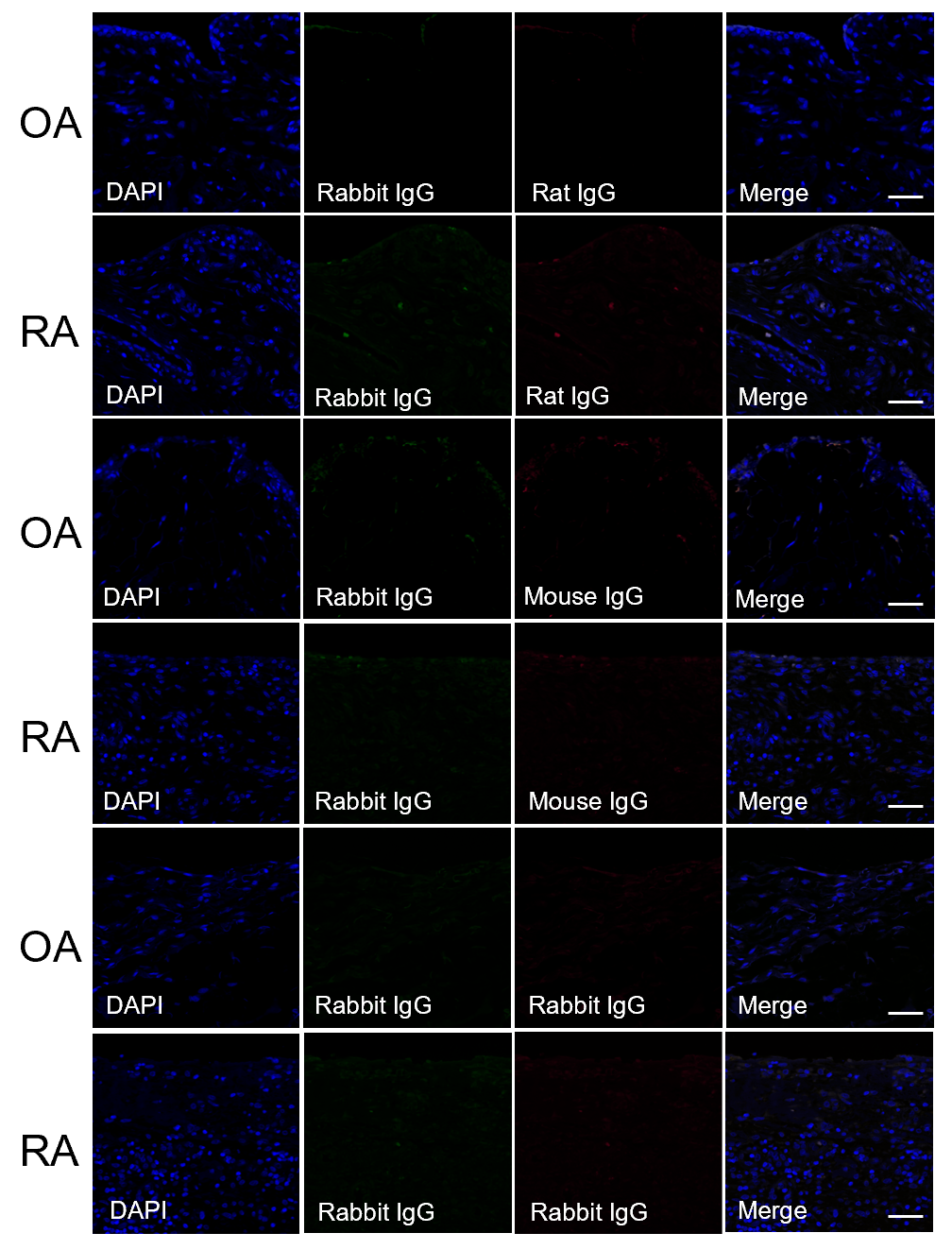


Figure S2: Multiplex immunohistochemical staining of antibody IgG isotype control for synovial tissues from patients with osteoarthritis (OA) or rheumatoid arthritis (RA). Scale bar, 50 μm.
